# Health and Fun

**Published:** 1870-10

**Authors:** 


### HEALTH AND FUN.

A correspondent writes : "My friends would call me a fool if, being in complete health, as I am, I were to adopt the rules in your book."

A healthy fool is happier than a sick Solomon. If a person has thirty or forty years to live, it is surely better to eat regularly the best things, and pass those years in a happy exemption from sickness and suffering, than to eat anything and everything, at any time and every time that suits our convenience and our whims. The man who eats heartily three times a day without any unpleasant reminder afterwards, is amply repaid for the effort it may at times cost him to have regular meals, or the self-denial which he may practice in waiting for his regular dinner-time. The great general rule for all, especially for women and those who live in-doors most of the time, is, eat nothing whatever between meals, have an interval of not less than five hours between each meal, and let the last meal of the day be about sun-down, and let it consist of some cold bread and butter and a cup of hot tea. It is almost impossible for a person to follow this for a week, and not find an increasing relish for breakfast, to be thoroughly digested by the exercise of the hours following. Hearty suppers insure a want of appetite for breakfast. The book on "Good Living" is full on this point.

